# Dental pulp stem cell‐derived exosomes alleviate cerebral ischaemia‐reperfusion injury through suppressing inflammatory response

**DOI:** 10.1111/cpr.13093

**Published:** 2021-07-07

**Authors:** Song Li, Lihua Luo, Yan He, Ruohan Li, Yangfan Xiang, Zhenjie Xing, Yejian Li, Abdullkhaleg Ali Albashari, Xiangyan Liao, Keke Zhang, Liang Gao, Qingsong Ye

**Affiliations:** ^1^ Department of Neurosurgery The Affiliated Changzhou No. 2 People's Hospital of Nanjing Medical University Changzhou China; ^2^ School and Hospital of Stomatology Wenzhou Medical University Wenzhou China; ^3^ Lab of Regenerative Medicine Tianyou Hospital Wuhan University, of Science and Technology Wuhan China; ^4^ Centre of Regenerative Medicine Renmin Hospital of Wuhan University Wuhan University Wuhan China; ^5^ Department of Shanghai Tenth People's Hospital Clinical Medical College Nanjing Medical University Nanjing China; ^6^ Department of Neurosurgery Shanghai Tenth People's Hospital Tongji University Shanghai China

**Keywords:** cerebral ischaemia‐reperfusion injury, dental pulp stem cells, exosomes, neuroinflammation, oxygen‐glucose deprivation–reperfusion

## Abstract

**Objectives:**

The study aimed to determine whether dental pulp stem cell‐derived exosomes (DPSC‐Exos) exert protective effects against cerebral ischaemia‐reperfusion (I/R) injury and explore its underlying mechanism.

**Materials and Methods:**

Exosomes were isolated from the culture medium of human DPSC. Adult male C57BL/6 mice were subjected to 2 hours transient middle cerebral artery occlusion (tMCAO) injury followed by 2 hours reperfusion, after which singular injection of DPSC‐Exos via tail vein was administrated. Brain oedema, cerebral infarction and neurological impairment were measured on day 7 after exosomes injection. Then, oxygen‐glucose deprivation–reperfusion (OGD/R) induced BV2 cells were studied to analyse the therapeutic effects of DPSC‐Exos on I/R injury in vitro. Protein levels of TLR4, MyD88, NF‐κB p65, HMGB1, IL‐6, IL‐1β and TNF‐α were determined by western blot or enzyme‐linked immunosorbent assay. The cytoplasmic translocation of HMGB1 was detected by immunofluorescence staining.

**Results:**

DPSC‐Exos alleviated brain oedema, cerebral infarction and neurological impairment in I/R mice. DPSC‐Exos inhibited the I/R‐mediated expression of TLR4, MyD88 and NF‐κB significantly. DPSC‐Exos also reduced the protein expression of IL‐6, IL‐1β and TNF‐α compared with those of the control both in vitro and in vivo. Meanwhile, DPSC‐Exos markedly decreased the HMGB1 cytoplasmic translocation induced by I/R damage.

**Conclusions:**

DPSC‐Exos can ameliorate I/R‐induced cerebral injury in mice. Its anti‐inflammatory mechanism might be related with the inhibition of the HMGB1/TLR4/MyD88/NF‐κB pathway.

## Graphical abstract

Dental pulp stem cell‐derived exosomes (DPSC‐Exos) alleviated cerebral ischemia‐reperfusion (I/R)‐induced brain oedema, cerebral infarction and neurological impairment in mice. DPSC‐Exos meliorated neuroinflammation after cerebral I/R and oxygen‐ and glucose‐deprived BV2 microglia via the HMGB1/TLR4/MyD88/NF‐κB pathway.

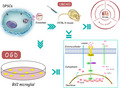

## 1 INTRODUCTION

Cerebral ischaemia can result in neuron damage, cognitive dysfunction, learning and memory disorders, neural function defects and even brain death. Ischaemic stroke is associated with substantial morbidity, mortality and disability.^1^ However, effective clinical treatments that can significantly improve the prognosis of ischaemic stroke are still unavailable. It is shown that mesenchymal stem cells (MSCs)‐based therapies are appealing in the treatment of ischaemic stroke.^2^, ^3^, ^4^ The obtaining process of these MSCs, such as the human bone marrow‐derived MSCs (BMSCs) and human adipose tissue‐derived MSCs, often involves invasive and painful procedures.^5^, ^6^ In comparison, dental pulp stem cells (DPSCs), originated from neural crest and enclosed in a dental pulp chamber, seem to be an excellent source of stem cells. Because DPSCs can be extracted from discarded teeth, which is non‐invasive and raises no ethic concerns.^7^, ^8^ A recent animal study showed that exosomes secreted from DPSCs had stronger immuno‐modulating effects than those from the BMSCs.^9^ An intravenous administration of DPSCs was proved to confer a similar functional recovery and superior reduction in infarct size following the middle cerebral artery occlusion in a rat model compared with the administration of BMSCs.^10^ As accumulating evidence indicated the potential of DPSCs in cell‐based therapy for neurological diseases and cardiac disease,^11^, ^12^, ^13^ DPSC has become an attractive candidate for ischaemic stroke therapy.

Animal studies and preclinical trials suggested that the therapeutic effects of stem cells were mainly attributed to the release of paracrine factors rather than the differentiation of stem cells.^14^, ^15^, ^16^ A study showed that intranasal administration of conditioned medium of stem cells from human exfoliated deciduous tooth facilitated the recovery of focal cerebral ischaemia. As one of the most pivotal paracrine mediators, stem cell‐derived exosomes showed therapeutic effects on ischaemic stroke, such as angiogenesis, neurogenesis, anti‐neuroinflammation and neuronal remodelling in brain.^17^, ^18^, ^19^ However, whether DPSC‐derived exosomes (DPSC‐Exos) having any therapeutic effect on the brain after ischaemic stroke remains unclear.

Recent research has suggested that exosomes are nanoscale vesicles, which could be generated from various types of stem cells, constitutively released to the extracellular milieu.^20^, ^21^, ^22^ Exosomes can facilitate the exchange of biomolecules such as lipids, proteins and genetic materials.^23^ Exosomes derived from mesenchymal stem cells showed diverse immunomodulatory properties in neurological disorders. For example, in a swine model of traumatic brain injury, animals treated by BMS‐derived exosomes had lower levels of nuclear factor‐kappa B (NF‐κB) and inflammatory markers (IL‐1, IL‐6, IL‐8 and IL‐18) compared with those in the control.^24^ In the treatment of subarachnoid haemorrhage of rats, BMSC‐derived exosomes obviously declined the levels of inflammatory proteins, such as high‐mobility group box 1 protein (HMGB1), toll‐like receptor‐4 (TLR4) and tumour necrosis factor‐α (TNF‐α).^25^

HMGB1 is a nuclear non‐histone DNA‐binding protein.^26^ It is released into cytoplasm and extracellular space in many inflammatory diseases, involving in the pathogenesis of cerebral ischaemia.^27^ It has also been shown that the level of HMGB1 circulating in blood was positively correlated with the severity and infarct volume in patients with ischaemic stroke.^28^ TLR4 links to HMGB1 and reflects the inflammatory response of the injured.^29^ TLR4 signalling pathway includes the myeloid differentiation protein 88 (MyD88)‐dependent pathway and the MyD88‐independent pathway.^30^ Following stimulation, TLR4 signalling pathways are activated, consequently triggering the activation of downstream NF‐κB. NF‐κB contains a family of transcription factors, which are composed of five different proteins: p50, p52, c‐Rel, RelB and p65. The classic pathway leads to the liberation of the p65/p50 heterodimer and the translocation to nucleus, where the p65/p50 complexes bind to target sites and induce the generation and release of various cytokines including IL‐6, IL‐1β and TNF‐α.^31^ These cytokines are critical players in the inflammatory cascade, leading to severe cerebral injury after ischaemic stroke. Therefore, the inhibition of cytoplasmic translocation of HMGB1 and TLR4 signal becomes a potential strategy treating the ischaemic stroke.^32^

Based on the aforementioned findings, we speculated that DPSC‐Exos might play a protective effect in cerebral ischaemia‐reperfusion‐induced brain injury. This study aimed to investigate the protective effect of DPSC‐Exos on I/R injured mouse brain and explore whether HMGB1/TLR4/MyD88/NF‐κB pathway was involved in this process.

## 2 MATERIALS AND METHODS

### 2.1 Ethics and animals

All animal experiments were approved and supervised by the Animal Ethics Committee of Wenzhou Medical University and were performed according to the guidelines of the Chinese National Institutes of Health and the Animal Care. 80 male C57BL/6 mice (wild type, 10 weeks of age, weighing 23‐25 g) were purchased from the Animal Center of Chinese Academy of Science (Shanghai, China). All animals were housed with free access to water and food in breeding cages under 50% ± 3% relative humidity at 23 ± 2°C.

### 2.2 Isolation, culture and identification of DPSCs

Healthy impacted third molars were obtained from patients between 18‐30 years old at the Department of Oral and Maxillofacial Surgery, Stomatology Hospital of Wenzhou Medical University. Informed consent was acquired from individuals whose teeth were collected for this study. All experiments using these DPSCs were approved by the Ethics Committee of the School and Hospital of Stomatology, Wenzhou Medical University. DPSCs were isolated as described in our previous work.^33^ Briefly, 70% v/v ethanol was used to sanitize the teeth surface prior to the removal of pulp tissues. Pulp tissues were minced (about 1 × 1 × 1 mm) and washed five times with phosphate‐buffered saline (PBS) containing 2.5% streptomycin/penicillin (S/P, Gibco, USA). Then, the pulp tissue was digested with 3 mg/mL collagenase type I (Gibco) and 4 mg/mL Dispase (Sigma‐Aldrich) for 30 minutes at 37°C. This pulp cellular suspension was suspended and incubated with α‐modified Eagle's medium (α‐MEM; Gibco) containing 20% foetal bovine serum (FBS; Gibco) and 1% S/P in 5% CO_2_ at 37°C. The medium was changed on day 6 and every 3 days thereafter. DPSCs in passages 3‐5 were used in this study.

Flow cytometry was performed to identify DPSCs as previously described.^33^ Typical positive and negative surface markers of MSCs, including human CD73 (BD Pharmingen™), CD34 (BioLegend), CD90 (BD Pharmingen), CD105 (BD Pharmingen), CD14 (BioLegend), CD45 (BioLegend), CD19 (BioLegend) and HLA‐DR (BioLegend) were evaluated according to manufacturers' protocols. The data were analysed by FlowJo 10 (Tree Star Inc.).

### 2.3 Isolation and identification of DPSC‐Exos

1 × 10^4^ cell/mL DPSCs were seeded in a T‐150 flask and cultured till 80% confluence. Cells were washed with PBS thrice and cultured in α‐MEM medium without FBS for 48 hours. An Exosome Isolation and Purification Kit (Umibio) was used to isolate the exosomes secreted by DPSCs in culture medium. To remove the cellular component, this 48‐hour‐old culture medium was centrifuged at 3000 *g* for 10 minutes at 4°C. Then, exosome concentration solution was added to the supernatants (1:4) and refrigerated at 4°C for 2 hours. Subsequently, the samples were centrifuged at 10 000 *g* (Beckman Coulter) for 1 hour at 4°C. The pelleted exosomes were resuspended in PBS and stored at −80°C. Protein concentration of the exosomes was quantified by a BCA protein assay kit (Beyotime). Surface marker proteins of the exosomes, including CD63, CD9 and CD81, were detected by western blot. The morphology and size of exosomes were measured by a transmission electron microscope (TEM, H‐7500; HITACHI) and NanoSight Tracking Analysis (Malvern NanoSight NS300).

### 2.4 Animal model of focal cerebral ischaemia and reperfusion

Animals were subjected to focal cerebral ischaemia by transient middle cerebral artery occlusion (tMCAO) at the right side and followed by reperfusion as described previously.^34^ Briefly, mice were anaesthetized by 2% isoflurane N_2_/O_2_ (70%:30%) mixture and maintained with 1% isoflurane via a face mask. 1‐cm‐long midline incision was made on the skin of anterior neck area after sterilization. Under an operating microscope, right common carotid artery (CCA), external carotid artery (ECA) and internal carotid artery (ICA) were exposed through blunt dissection. Permanent ligations were made at the origin of ECA and CCA with 6‐0 silk sutures, and an arteriotomy was made in the CCA. To completely block the middle cerebral artery (MCA), a silicone‐coated 6‐0 nylon filament (L1800; Jialing Biotechnology) was inserted into the CCA and advanced over 9‐10 mm to the carotid bifurcation along the ICA and to the origin of the MCA. Two hours later, reperfusion was performed by removing the filament carefully and the incision of CCA was permanently ligated. The body temperature of the animals was maintained at 37 ± 0.5°C with a heating pad during surgery and anaesthesia recovery.

Two hours after reperfusion, animals administrated with DPSC‐Exo (10 µg total protein per 100 µL PBS^34^) via tail vein were marked as the I/R + DPSC‐Exos group; animals administrated with 100 µL PBS were marked as the I/R + PBS group; animals went through surgical procedures but avoided the placement of filament at MCA were marked as the sham group. Mice were randomly assigned to the study groups. Grouping information was disclosed until result interpretation.

### 2.5 Determination of brain oedema

Brain oedema of the mice was determined by a dry–wet method as described previously.^35^ In short, brain was sampled on day 7 after the surgical procedure (n = 6 per group) and weighed by an electronic balance as fresh and after being dried in an oven at 100°C for 48 hours. The percentage of water content in brain was calculated as follows:

(1) $$\text{The}\;\text{brain}\;\text{water}\;\text{content}\;\left(\%\right)=\frac{\text{wet}\;\text{weight}‐\text{dry}\;\text{weight}}{\text{wet}\;\text{weight}}\times 100\%,$$

Representing the level of brain oedema.

### 2.6 Infarct volume qualification

Brain infarct size was assessed using a 2,3,5‐triphenyltetrazolium chloride staining (TTC; Sigma‐Aldrich).^36^ On day 7, 6 mice/group were sacrificed. Mouse brain was dissected and frozen at −80°C for 10 minutes and sectioned into 2‐mm‐thick slices coronally. Brain slices were incubated in 2% TTC solution at 37°C for 15 minutes and fixed by 4% paraformaldehyde. Then stained slices were photographed with a digital camera (Canon). The infarct volume was measured and estimated by ImageJ software (National Institute of Health). The infarct volume of brain was calculated as follows:

(2) $$\text{The}\;\text{infarct}\;\text{volume}\;\left(\%\right)=\frac{\text{volume}\;\text{of}\;\text{white}\;\text{color}\;\text{brain}\;\text{mass}}{\text{volume}\;\text{of}\;\text{entire}\;\text{brain}\;\text{mass}}\times 100\%.$$

### 2.7 Neurological deficit assessment

To assess the neurological changes, modified neurological severity scores (mNSS) were performed by a skilful investigator blinded to the groups on day 7 (n = 18 per group). The motion, sensation and reflex of animals were evaluated.^37^ The score was graded on a scale of 0 to 14, where 0 represented no evident neurological deficit, and 14 represented severe deficit. The behavioural assessment was performed in a dedicated room with controlled temperature (22 ± 1°C).

### 2.8 Oxygen‐glucose deprivation study on murine BV2 microglia

Cellular hypoxia was constructed using a method described previously.^38^ Murine BV2 microglia cell line was purchased from YaJi Biological Company (Shanghai). During cellular maintaining, BV2 cells were cultured in high‐glucose Dulbecco's modified Eagle's medium (DMEM; Gibco) with 10% FBS and 1% S/P in a humidified ambience and 5% CO_2_ at 37°C. Half medium change was performed every 2 days. Cells were seeded into 96‐well plates (1 × 10^4^ cell/well for the cellular viability test and immunofluorescence staining) and 24‐well plates (1 × 10^5^ cell/well for the enzyme‐linked immunosorbent assay). Cells in logarithmic growth were washed three times with PBS and maintained in glucose‐free DMEM (Gibco). Subsequently, these cells were placed in an anaerobic environment for 4 hours using an AnaeroPack rectangular jar (Mitsubishi Gas Company). After 4 hours, cells were treated with fresh high‐glucose DMEM with 10% FBS and 1% S/P containing 40 µg/mL DPSC‐Exos and placed back in the 5% CO_2_ incubator for 24 hours. BV2 cells were randomly assigned to three groups including control, oxygen‐glucose deprivation (OGD) and OGD + DPSC‐Exos.

To evaluate the therapeutic effect of DPSC‐Exos on oxygen‐ and glucose‐deprived BV2 cells, cellular proliferation was assessed via a cell counting kit‐8 assay (CCK‐8; Beyotime),^39^ and the inflammatory proteins (HMGB1, IL‐6, IL‐1β and TNF‐α) were qualitatively and quantitatively studied through immunofluorescence staining and enzyme‐linked immunosorbent assay (ELISA).^40^

### 2.9 Enzyme‐linked immunosorbent assay

The concentrations of pro‐inflammatory cytokines (IL‐6, IL‐1β and TNF‐α) in mouse brain and BV2 cell supernatants were quantified by ELISA kits (Proteintech). The experimental procedure was performed strictly in accordance with the manufacturer's manual. The absorbance was obtained by a microplate reader at 450 nm. For the in vivo study, ipsilateral brain samples were obtained on day 7, 3 mice/group.

### 2.10 Immunofluorescence staining

To visualize the expression of HMGB1 on mouse brain and BV2 cells, immunofluorescence staining was performed as described in our previous research.^33^ In brief, after perfusion and fixation, the infarcted cerebral hemisphere (n = 3 per group) was prepared into paraffin sections. Brain sections (6 µm‐thickness) were sliced and blocked with 5% FBS at room temperature for 1 hour. BV2 cells were fixed in 4% paraformaldehyde for 15 minutes and washed twice with PBS. Then the cells were permeabilized by using 0.3% Triton‐100 in PBS at room temperature for 20 minutes. Thereafter, samples of brain sections and BV2 cells were incubated in rabbit anti‐HMGB1 antibody (1:100; Abcam) at 4°C overnight. After rinsed three times with PBS, samples were incubated with Alexa Flour 594 conjugated goat anti‐rabbit IgG (1:500; Proteintech) at room temperature for 1 hour. Cell nuclei were counter‐stained with DAPI (Beyotime) at room temperature for 3 minutes. All fluorescent images were captured and imaged by a fluorescence microscope (Carl Zeiss).

### 2.11 Total/nuclear protein extraction and western blot analysis

The total proteins, nuclear proteins or cytoplasmic proteins were extracted from the exosomes or cortex tissues of the infarcted cerebral hemisphere according to the manufacturer's instructions (Beyotime). The amount of protein was quantified by the BCA assay. Equal amounts of protein were electrophoresed by 10% SDS‐PAGE and then transferred to polyvinylidene difluoride (PVDF) membranes. After blocking, membranes were incubated with the following primary antibodies, including CD63 (1:500; Abcam), CD9 (1:500; Abcam), CD81 (1:500; Abcam), TLR4 (1:1000; Affinity Biosciences), NF‐κB p65 (1:1000; Affinity Biosciences), HMGB1 (1:1000; Abcam), GAPDH (1:2000; Affinity Biosciences), MyD88 (1:1000; Affinity Biosciences), β‐actin (1:2000; Proteintech), Histone H3 (1:2000; Affinity Biosciences) and the secondary HRP‐labelled antibodies (1:5000; Proteintech). Band visualization was enhanced with chemiluminescence (ECL; Biological Industries), and the results were processed with a Chemiluminescent Imaging and Analysis System (Biolight Biotechnology Co., Ltd.).

### 2.12 Statistical analysis

All data were obtained from at least three independent experiments and presented as mean ± standard deviation (SD). prism 6 (Graphpad Software) was used for graphic presentation. Statistical analysis was performed by using spss 22.0 (SPSS Inc.). For multiple group comparisons, one‐way ANOVA analysis was used, and followed by the LSD or Dunnett's T3 post hoc comparisons test. *P* < .05 was considered as the differences being statistically significant.

## 3 RESULTS

### 3.1 Identification of DPSCs and DPSC‐Exos

**FIGURE 1:**
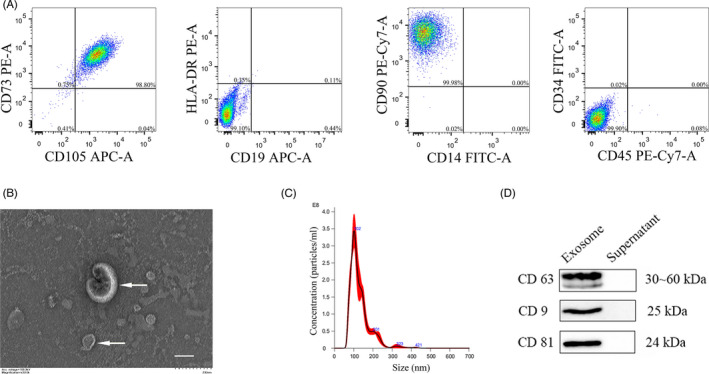
Identification of DPSCs and DPSC‐derived exosomes. (A) The expression of DPSCs surface antigens by flow cytometry. (B) Representative TEM image of DPSC‐Exos. White arrows indicated exosomes. (C) The diameter of DPSC‐Exos determined by NTA. (D) The expression of specific markers of exosomes (CD63, CD9 and CD81) in the vesicles from DPSCs by western blot. Scale bar = 100 nm. DPSC‐Exos, dental pulp stem cell‐derived exosomes; DPSCs, dental pulp stem cells; NTA, nanoparticle tracking analysis; TEM, transmission electron microscopy

The results of flow cytometry showed that DPSCs strongly expressed the MSCs markers, CD73, CD90 and CD105, but lacked the expression of the surface antigen of hematopoietic stem cells including CD14, CD19, CD34, CD45 and HLA‐DR (Figure 1A). TEM image demonstrated that the round‐shape morphology of DPSC‐Exos vesicles indicated by white arrows (Figure 1B), and we found that the diameter of these DPSC‐Exos vesicles was around 100 nm (Figure 1C). Besides, western blot indicated that the characteristic exosome surface markers (CD9, CD63 and CD81) were positive in DPSC‐Exos, which were isolated using the protocols introduced in this work (Figure 1D). Together, all analyses confirmed that DPSCs and DPSC‐Exos have been successfully isolated and identified.

### 3.2 DPSC‐Exos alleviated the brain oedema, cerebral infarction and neurological impairment in I/R mice

**FIGURE 2:**
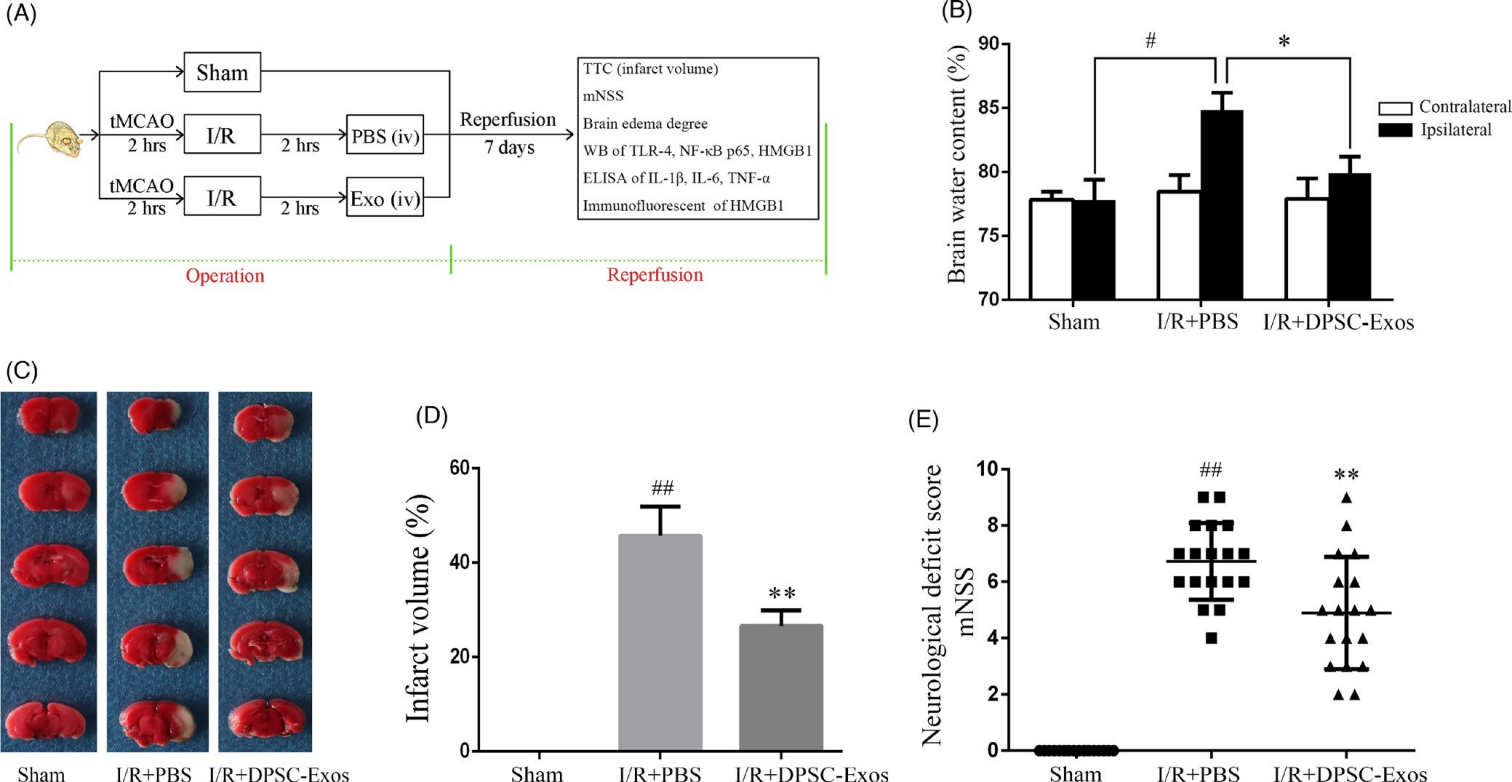
Neuroprotective effect of DPSC‐Exos on the mice with cerebral I/R injury. (A) Illustration of experimental procedure. (B) Brain water content reflecting brain oedema was assessed on day 7 after I/R injury (n = 6). (C) Representative images of brain slices, in which TTC staining was applied to differentiate infarction. (D) Quantitative analysis of the infarct volume (n = 6). (E) Neurological deficit was evaluated by a modified neurological severity scores (mNSS) (n = 18). Data were expressed as means ± SD. ^#^ *P* < .05 and ^##^ *P* < .01 versus the sham group; *P < .05 and **P < .01 versus the I/R + PBS group. DPSC‐Exos, dental pulp stem cell‐derived exosomes; I/R, ischaemia/reperfusion; mNSS, modified neurological severity score; PBS, phosphate‐buffered saline; tMCAO, transient middle cerebral artery occlusion; TTC, 2,3,5‐Triphenyltetrazolium chloride; WB, western blot

The effects of DPSC‐Exos on the brain oedema, cerebral infarction and neurological impairment have been tested in I/R mice according to the proposed protocol (Figure 2A). The ipsilateral brain water content in the I/R + PBS group was (84.84 ± 1.35) %, much higher than that of the (77.82 ± 1.58) % in the sham group (*P* < .05), suggesting an impactful ischaemic brain damage was achieved by the methods applied on mice (Figure 2B). Moreover, single IV injection of DPSC‐Exos could remarkably alleviated the brain oedema (*P* < .01), where the water content in the DPSC‐Exos group remained at the same level as in the sham group (Figure 2B). TTC‐stained brain slices revealed that the infarct volume was significantly (*P* < .01) decreased from (45.70 ± 6.22) % in the I/R + PBS group to (28.18 ± 3.85) % in the I/R + DPSC‐Exos group (Figure 2C,D). The neurological deficiency scores decreased significantly in DPSC‐Exos‐injected mice compared with those in the I/R + PBS group (Figure 2E). These data demonstrated that DPSC‐Exos administration resulted in a favourable neurological recovery after I/R in mice.

### 3.3 DPSC‐Exos inhibited the I/R‐mediated TLR4 and NF‐κB activation

**FIGURE 3:**
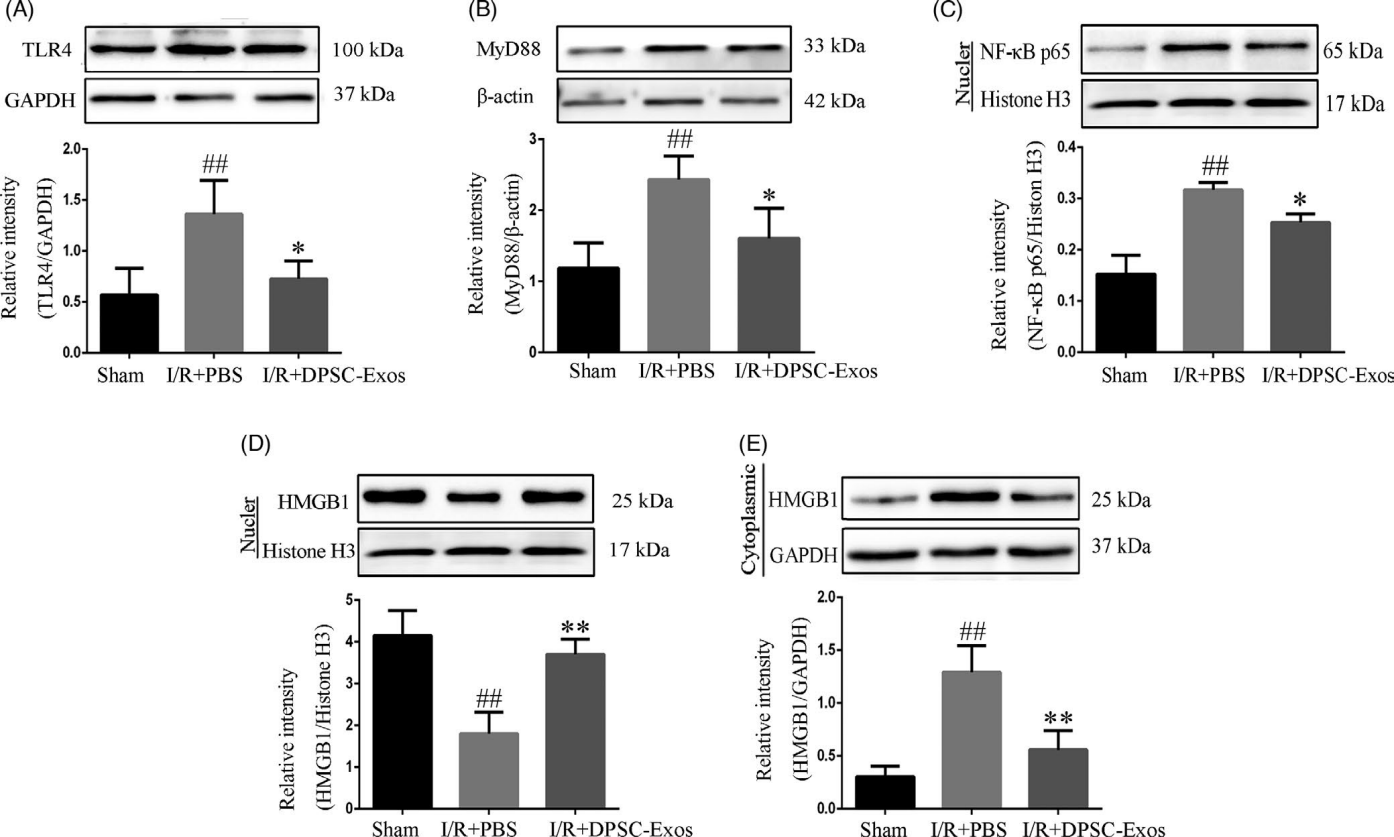
Effect of DPSC‐Exos on the expression of TLR4, MyD88, NF‐κB p65 and HMGB1 on day 7 after cerebral I/R damage. (A) The relative expression level of TLR4. (B) The relative expression level of MyD88. (C) The relative expression level of NF‐κB p65. (D) The relative expression level of nuclear HMGB1. (E) The relative expression level of cytoplasmic HMGB1. Protein samples were acquired from the ischaemic cortex and assayed by western blot. Nuclear proteins were normalized to the intensity of Histone H3, and cytoplasmic and total proteins were normalized to the intensity of GAPDH or β‐actin. Data were expressed as means ± SD (n = 3). ^##^ *P* < .01 versus the sham group; *P < .05 and **P < .01 versus the I/R + PBS group. DPSC‐Exos, dental pulp stem cell‐derived exosomes; HMGB1, high‐mobility group box 1 protein; I/R, ischaemia/reperfusion; MyD88, myeloid differentiation protein 88; NF‐κB, nuclear factor‐kappa B; PBS, phosphate‐buffered saline; TLR4, toll‐like receptor‐4

Western blot analysis was employed to identify the expression of TLR4, MyD88 and NF‐κB p65. Ischaemic damage increased the level of TLR4 in the infarcted cerebral hemisphere (*P* < .01), while DPSC‐Exos injection notably downregulated the TLR4 expression (*P* < .05) (Figure 3A). The intensity of MyD88 upregulated obviously upon I/R stimulation (*P* < .01), while DPSC‐Exos administration effectively suppressed the expression of I/R‐induced MyD88 (*P* < .05) (Figure 3B). The intensity of nuclear NF‐κB p65 was extremely upregulated because of ischaemic damage (*P* < .01), while DPSC‐Exos suppressed the expression of NF‐κB p65 significantly compared with the I/R + PBS group (*P* < .05) (Figure 3C).

### 3.4 Effects of DPSC‐Exos on survive of BV2 cells

**FIGURE 4:**
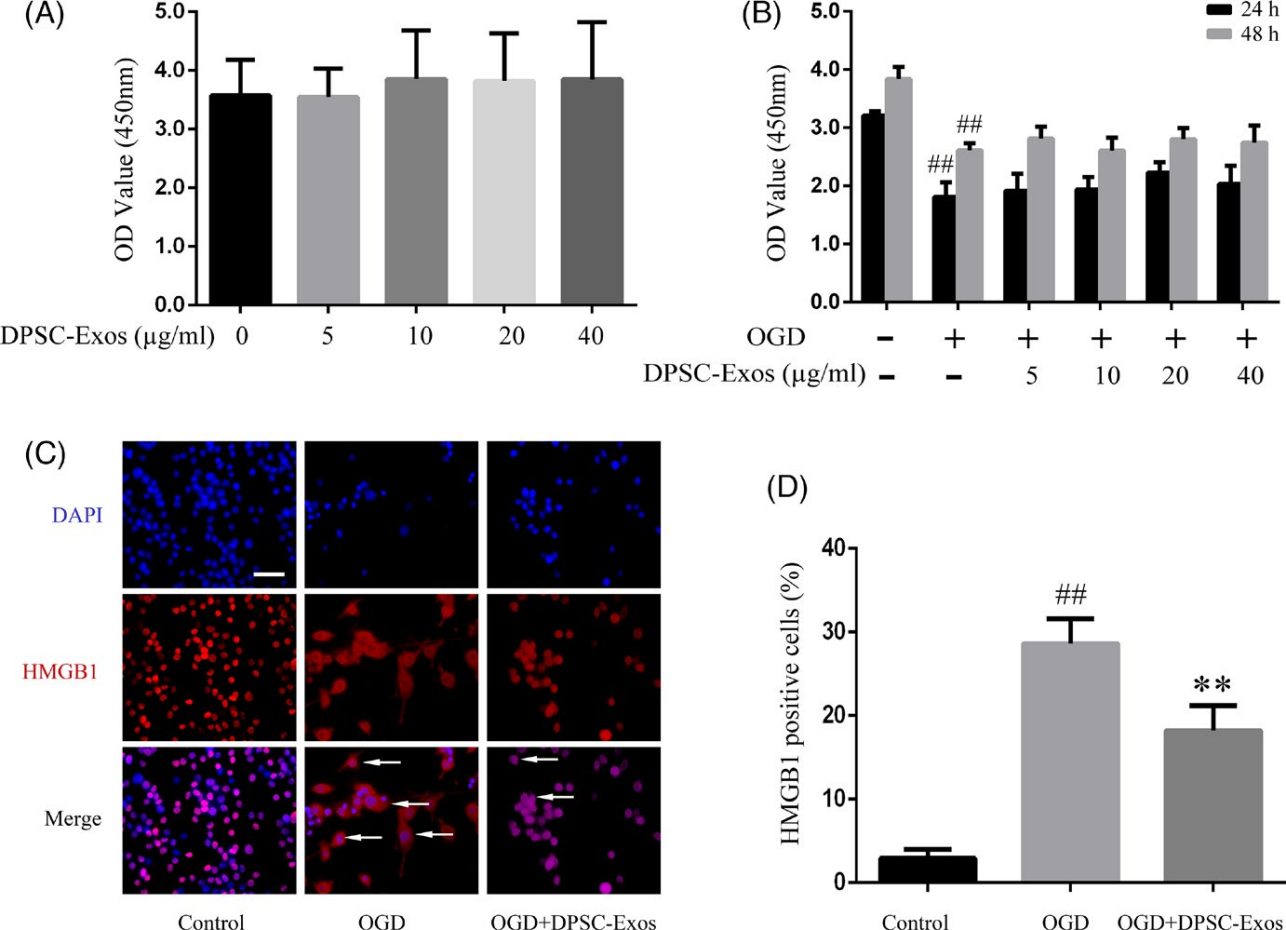
Effect of DPSC‐Exos on cell viability and HMGB1 cytoplasmic translocation of BV2 cells with OGD damages. (A) Cell viability of BV2 cells subjected to DPSC‐Exos of various concentrations (5, 10, 20 and 40 μg/mL) as assayed by CCK‐8 assay with the absorbance being read at 450 nm. (B) Cell proliferation of BV2 cells with 4 h OGD followed by 24‐h and 48‐h reperfusion with DPSC‐Exos of various concentrations (5, 10, 20 and 40 μg/mL). (C) Representative immunofluorescence images of HMGB1‐positive cells (red, cytoplasm expression) in BV2 microglia. Cell nuclei were stained by DAPI as blue. White arrows indicated HMGB1‐positive cells. (D) Comparison of the HMGB1‐positive cells. ^##^ *P* < .01 versus the control group; **P < .01 versus the OGD group. All data were expressed as means ± SD (n = 3). Scale bar = 50 µm. DPSC‐Exos, dental pulp stem cell‐derived exosomes; HMGB1, high‐mobility group box 1 protein; I/R, ischaemia/reperfusion; OGD, oxygen‐glucose deprivation–reintroduction

The biosafety and activity of DPSC‐Exos on BV2 cells was determined by the CCK‐8 assay. No significant changes were observed in the viability of BV2 cells when treated with DPSC‐Exos at varied concentrations (5, 10, 20, 40 µg/mL) for 24 hours (Figure 4A). When BV2 cells were deprived of oxygen and glucose, the proliferation of BV2 was hampered (*P* < .01) and the DPSC exosomes (5, 10, 20, 40 µg/mL) did not affect the proliferation of these OGD‐challenged cells at 24 and 48 hours (Figure 4B). Among all concentrations studied, although 20 µg/mL of DPSC‐Exos showed a slight increase in the CCK‐8 value, there was no significant difference between 20 and 40 µg/mL of DPSC‐Exos on the cell proliferation after OGD both at 24 and 48 hours (*P* > .05) (Figure 4B). Literature indicated that exosomes showed a concentration‐dependent effect on the anti‐inflammatory activation of BV2 cells.^41^ Therefore, we selected 40 µg/mL of DPSC‐Exos to perform the following experiments.

### 3.5 DPSC‐Exos attenuated the neuroinflammation of post‐I/R and post‐OGD/R

**FIGURE 5:**
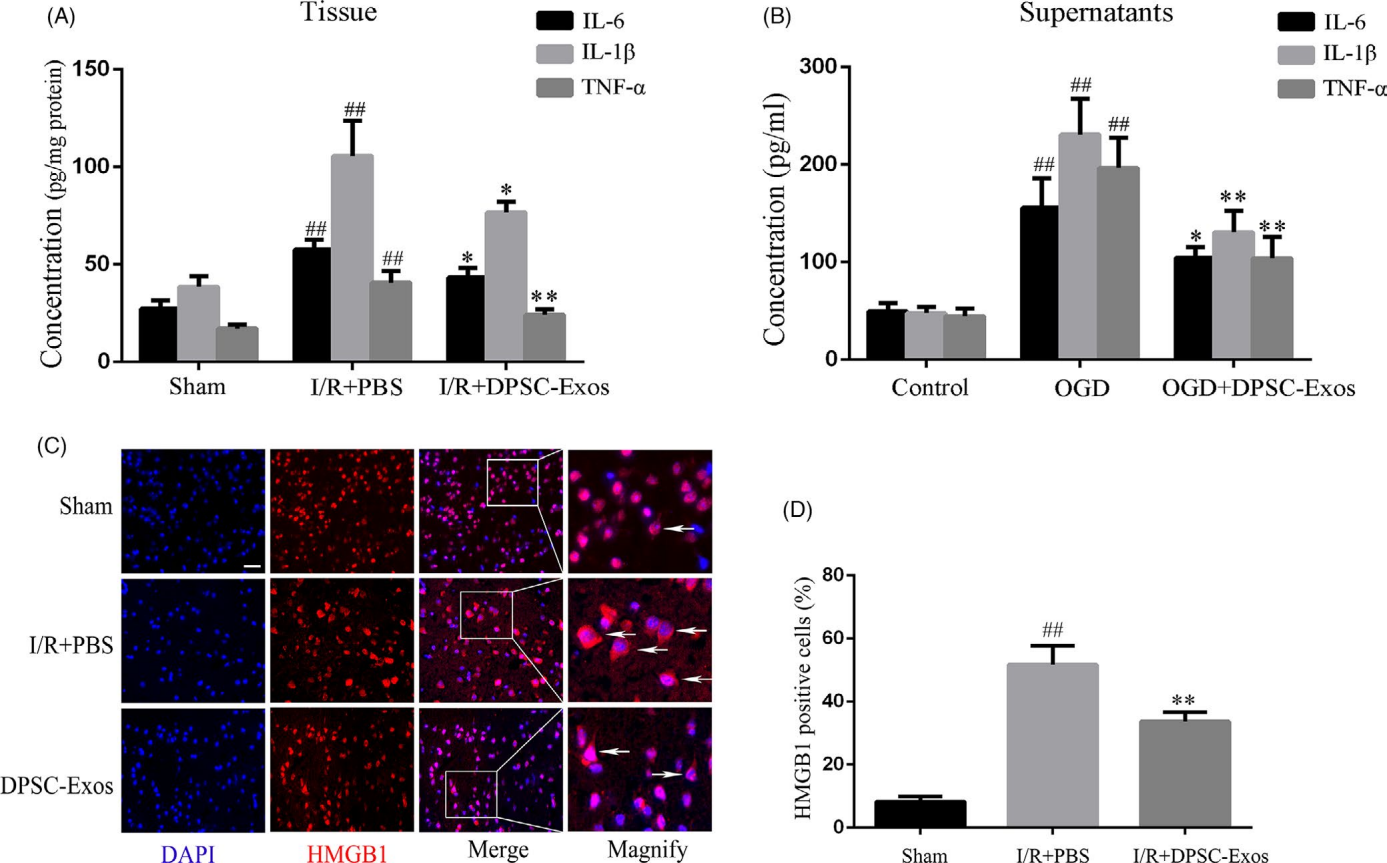
Effects of DPSC‐Exos on the release of inflammatory cytokines and the cytoplasmic translocation of HMGB1 in I/R injured mice and OGD‐challenged BV2 cells. (A) The levels of the pro‐inflammatory cytokines IL‐6, IL‐1β and TNF‐α in the ischaemic cerebral hemisphere as assayed by ELISA on day 7 after reperfusion. ^##^ *P* < .01 versus the sham group; *P < .05 and **P < .01 versus the I/R group. (B) The levels of pro‐inflammatory cytokines IL‐6, IL‐1β and TNF‐α in supernatant of BV2 cells after OGD damage as assayed by ELISA at 24 h after 4‐h deprivation of oxygen and glucose. ^##^ *P* < .01 versus the control group; *P < .05 and **P < .01 versus the OGD group. (C) Representative immunofluorescence images of HMGB1‐positive cells (red, cytoplasm expression) on I/R brain. Cell nuclei were stained by DAPI as blue. White arrows indicated HMGB1‐positive cells. (D) Comparison of the HMGB1‐positive cells. ^##^ *P* < .01 versus the sham group; **P < .01 versus the I/R + PBS group. Data were expressed as means ± SD (n = 3). Scale bar = 50 µm. DPSC‐Exos, dental pulp stem cell‐derived exosomes; ELISA, enzyme‐linked immunosorbent assay; HMGB1, high‐mobility group box 1 protein; I/R, ischaemia/reperfusion; IL, interleukin; OGD, oxygen‐glucose deprivation–reintroduction; PBS, phosphate‐buffered saline; TNF, tumour necrosis factor

To assess the effect of DPSC‐Exos on the secretion of neuroinflammation cytokines, ELISA was employed to determine the expression of the pro‐inflammatory cytokines (IL‐6, IL‐1β and TNF‐α) of the infarcted brain cortex of the mice and the supernatant of oxygen‐ and glucose‐deprived BV2 cells. Compared with the sham group, the level of inflammatory cytokines IL‐6, IL‐1β and TNF‐α was much higher in the I/R + PBS injury (*P* < .01) (Figure 5A). In contrast with the I/R + PBS group, the expression of these cytokines was significantly decreased in the I/R + DPSC‐Exos group (*P* < .05, *P* < .05 and *P* < .01, respectively). Similar observation was observed in in vitro study, and under the deprivation of oxygen and glucose, the expression of IL‐6, IL‐1β and TNF‐α was significantly increased in the supernatant of BV2 cells in the OGD group (*P* < .01) (Figure 5B). With the addition of DPSC‐Exos, they were greatly decreased in the OGD + DPSC‐Exos group compared with those in the OGD group (*P* < .05, *P* < .01 and *P* < .01, respectively).

### 3.6 DPSC‐Exos inhibited the HMGB1 cytoplasmic translocation in vivo and in vitro

The nuclear intensity of HMGB1 decreased dramatically in the I/R + PBS mice compared with that in the sham group (*P* < .01), while DPSC‐Exos injection inhibited the I/R‐mediated HMGB1 release effectively (*P* < .01) (Figure 3D). The cytoplasmic intensity of HMGB1 was upregulated obviously upon I/R stimulation (*P* < .01), while DPSC‐Exos addition effectively suppressed the cytoplasmic translocation of HMGB1 in I/R‐induced cells (*P* < .01) (Figure 3E). In addition, immunofluorescence staining was adopted to explore the effect of DPSC‐Exos on the translocation of HMGB1 at the ischaemic brain cortex and in the oxygen‐ and glucose‐deprived BV2 cells. There were more HMGB1‐positive cells in the OGD group, expressed in cytoplasm, than in the sham group (*P* < .01) (Figure 4C,D). After the application of DPSC‐Exos, the number of HMGB1‐positive cells was remarkably reduced compared with that in the OGD group. In animal study, cytoplasmic translocation of HMGB1 was extremely elevated in the I/R + PBS group compared with the sham group (*P* < .01). In contrast to the I/R + PBS group, DPSC‐Exos administration remarkably suppressed the cytoplasmic translocation of HMGB1 and restored its nuclear staining at the cortex region of infarcted cerebral hemisphere (*P* < .01) (Figure 5C,D).

## 4 DISCUSSION

In our study, we investigated whether DPSC‐Exos had a potential protective effect on cerebral I/R injury and how it affected. The main findings were summarized as follows: (1) A single systemic administration of 10 µg cell‐free DPSC‐Exos could significantly attenuate the cerebral infarction, brain oedema and neurological impairment in I/R mice; (2) DPSC‐Exos could alleviate the neuroinflammation of ischaemic challenged brain and oxygen‐ and glucose‐deprived BV2 cells; (3) HMGB1/TLR4/MyD88/NF‐κB pathway was activated by cerebral I/R, and DPSC‐Exos inhibited the neuroinflammatory reaction via the HMGB1/TLR4/MyD88/NF‐κB pathway in cerebral I/R mice. These findings were the first study reporting that DPSC‐Exos could provide neuroprotection against cerebral I/R‐induced neuroinflammation through inhibiting the HMGB1/TLR4/MyD88/NF‐κB signalling pathway.

Accumulating evidences indicated that inflammatory response played a key role in the pathogenesis of cerebral I/R injury, and the anti‐inflammatory treatment might be significant in ischaemic stroke.^42^, ^43^, ^44^ Pro‐inflammatory cytokines could not only damage peripheral neural cells, but destroy the permeability of the blood brain barrier which would aggravate the cerebral oedema and further cause brain damage after an ischaemic stroke.^45^, ^46^, ^47^ In accordance with previous reports,^48^, ^49^ we found that the release of pro‐inflammatory cytokines increased the cerebral I/R injury in mice. Importantly, our study showed that the inflammation was significantly suppressed by DPSCs‐Exos treatment. Stem cell‐based therapies were used in clinical trials for ischaemic stroke and other neurological diseases, and there was robust literature confirming the therapeutic effect of stem cell‐based treatments for stroke.^50^, ^51^ However, direct application of stem cells remains a few issues like potential side effects, including tumourigenicity, immunosuppression, pulmonary embolism^52^ and insufficient number of stem cells passing through the blood brain barrier. Due to the small size, exosomes could easily pass the blood brain barrier, confirmed in a murine model.^53^ The application of exosomes might minimize the potential adverse effects of the direct use of stem cells.^54^ Here, we provided new evidence that cell‐free DPSC‐Exos showed therapeutic effect treating ischaemic stroke in mice.

Studies suggested that exogenous stem cell‐derived exosomes given to rodents could maintain immune privilege, causing no immune response and no harm to healthy animals. In a previous study, there were no abnormal histopathological findings in brain, heart, lung, liver and kidney after intravenous injection of stem cell‐derived exosomes in healthy rats.^55^ Another study reported that DiI‐labelled exosomes distributed not only in brain tissue, but in peripheral organs of lung, liver and spleen after 24 hours of tail vein administration in a rat model of intracerebral haemorrhage. They also found co‐labelling among DiI, NeuN (a marker of neuron), Iba‐1 (a marker of microglia) and CNP‐ase (a marker of oligodendrocyte) after injection of exosomes.^53^ Several studies confirmed that DPSCs were superior to BMSCs in neuroprotection and migration after ischaemic stroke.^9^, ^10^, ^56^ However, whether DPSC‐Exos have any advantage in treating ischaemic stroke is still unknown. In our study, single dose of 10 µg total protein of DPSC‐Exos was injected via tail vein on I/R‐challenged mice and its therapeutic effect was evaluated on day 7. The infarct volume in I/R + DPSC‐Exos group was reduced by 38.34% compared with the I/R + PBS group. This finding was consistent with the literature, where around 30% reduction was reported in the application of either stem cells or exosomes in rescuing ischaemic rats.^54^, ^57^, ^58^

After stroke, cytoplasmic translocation and the release of HMGB1 would stimulate neurocyte and cause severe inflammatory response through signalling and molecular transport mechanisms.^59^ Similar to previous study,^27^ our research showed that cerebral I/R damage drastically activated the HMGB1 signalling, promoting the expression of TLR4, MyD88, and NF‐κB (Figure 3A–E, Figure 5C,D). Many researches indicated that the activation of HMGB1/TLR4/MyD88/NF‐κB pathway was associated with the aggravation of cerebral I/R damage.^28^, ^60^ Strikingly, our study showed that DPSC‐Exos treatment suppressed the cytoplasmic translocation of HMGB1 both in vivo and in vitro and inhibit the in vivo expression of TLR4, MyD88 and NF‐κB. Therefore, we speculated that the neuroprotective mechanism of DPSC‐Exos might relate to the suppression of HMGB1/TLR4/MyD88/NF‐κB signalling pathway. Additionally, because microglia‐derived inflammatory cytokines could adversely activate downstream signalling pathways and cause harmful reactions, including destruction of neuronal function and direct neurotoxicity.^61^ We adopted a microglia cell line, BV2, to establish an oxygen‐ and glucose‐deprived model, OGD, to further verify the anti‐inflammatory effect of DPSC‐Exos in vitro. In this in vitro study, DPSC‐Exos inhibited the cytoplasmic translocation of HMGB1 in BV2 cells subjected to OGD. We observed an anti‐inflammatory effect of DPSC‐Exos in BV2 cells under OGD, where the expression of IL‐6, IL‐1β and TNF‐α was decreased by DPSC‐Exos at the dose of 40 µg/mL. We assumed that the anti‐inflammatory effect of DPSC‐Exos in BV2 cells might be correlated with the regulation of HMGB1 activity. Those findings were in consistence with the previous studies on MSCs‐derived exosomes, which displayed an anti‐inflammatory effect of exosomes in ischaemic stroke.^19^, ^55^, ^58^ An in vitro study reported that BMSCs‐derived exosomes increased the viability of rat pheochromocytoma (PC12) cells in OGD.^62^ In current study, we found that DPSC‐Exos was not toxic to the BV2 cells. Yet it had no influence on the cell proliferation after OGD. We discovered that the protection of DPSC‐Exos over OGD‐challenged cells lied in its anti‐inflammatory effect. However, we speculated that cell proliferation, in the long term, shall benefit from this DPSC‐Exos as it reduced inflammatory cytokines and avoid cell damage. This has been echoed in our in vivo study, where the expression of the same inflammatory cytokines was inhibited, and brain damage was confined after a single administration of DPSC‐Exos.

In summary, our study determined that DPSCs‐Exos contributed to the inhibition of HMGB1/TLR4/MyD88/NF‐κB pathway and alleviated the cerebral I/R damage (including brain oedema, cerebral infarction and neurological deficit) in mice after tMCAO. This study provided preclinical evidence for further use of DPSC‐Exos in cerebral I/R therapy to improve the treatment outcome of patients who suffered from ischaemic stroke.

## CONFLICT OF INTEREST

The authors declare no conflict of interest.

## AUTHOR CONTRIBUTIONS

Liang Gao, Song Li, Lihua Luo and Qingsong Ye contributed to the initial experimental discussion and designs. Song Li, Yangfan Xiang and Zhenjie Xing set up the tMCAO and OGD model, analysed the data, and Song Li, Yejian Li, Abdullkhaleg Albashari and Xiangyan Liao performed experiments. Song Li, Yan He, Ruohan Li, Keke Zhang and Qingsong Ye wrote or revised the manuscript. All authors have reviewed the final manuscript and approved the submission to this journal.

## Data Availability

The data sets used in this study are available from corresponding authors on a reasonable request.
